# Safety and efficacy of 27-gauge transconjunctival vitrectomy for the diagnosis of posterior uveitis or pan uveitis of unknown origin

**DOI:** 10.1186/s12886-022-02405-y

**Published:** 2022-04-19

**Authors:** Atsushi Sakai, Mizuki Tagami, Norihiko Misawa, Manabu Yamamoto, Takeya Kohno, Shigeru Honda

**Affiliations:** Department of Ophthalmology and Visual Sciences, Graduate School of Medicine, Osaka City University, Osaka, Japan

## Abstract

**Background:**

Diagnostic vitrectomy is an important method for evaluating uveitis, and its diagnostic utility is high regardless of whether the uveitis is infectious or non-infectious. The course of diagnostic vitreous surgery with 27-gauge pars plana vitrectomy and perioperative complications is reported.

**Methods:**

An observational retrospective study of patients who underwent 27-gauge diagnostic vitrectomy due to atypical intraocular inflammation was conducted. The final diagnosis rate, complications due to surgery, preoperative visual acuity, and postoperative visual acuity (1 month and 6 months after surgery) were examined retrospectively.

**Results:**

Diagnostic vitreous surgery was performed in 32 patients and 35 eyes (14 males and 18 females, age 14–85 years, median 67 years) during the study period. The average operation time was 52 min for 19 eyes with cataract surgery and 35 min for 16 eyes without cataract surgery. Preoperative log(minimum angle of resolution [MAR]) visual acuity was 0.84 ± 0.87, 1-month postoperative logMAR visual acuity was 0.41 ± 0.55 (*p* = 0.004, *n* = 28), and 6-month postoperative average logMAR visual acuity was 0.45 ± 0.73 (*p* = 0.012, *n* = 15). The diagnosis was made by diagnostic vitrectomy in 19 cases (54%). Postoperative complications were observed in 2 of 35 postoperative patients (5%); one involved increased intraocular pressure, and the other case involved vitreous hemorrhage of the eye, necessitating reoperation.

**Conclusion:**

Diagnostic 27-gauge vitrectomy could be effective for evaluating intraocular inflammation.

## Introduction

Uveitis is divided into infectious and non-infectious, and there is a definition based on the site of occurrence [1–5]. Of them, posterior uveitis and pan uveitis, which cause significant problems in visual function, require accurate diagnosis [6–9]. In addition, even in the pathological condition that is being followed up as vitreous opacification in general medical care, very important diseases such as intraocular lymphoma may be hidden, and careful diagnosis is required [10–12].

Vitreous opacity is generally an age-associated change, but it can sometimes be caused by various illnesses affecting the whole body. For example, vitreous opacity can be associated with genetic predisposition, inflammatory infectious or non-infectious conditions, and degenerative, traumatic, or idiopathic causes [13].

We can determine the cause of vitreous opacity through ophthalmic examinations, medical examinations, laboratory tests, and image analysis, but because these approaches are limited, it may not be possible in some cases to identify the cause of the findings. One alternative for evaluating ophthalmic phenomena is diagnostic vitrectomy, which has been performed for a long time. In particular, the use of 25-gauge transconjunctival vitrectomy is increasing [14–16].

Several reports have described the safety and efficacy of 25-gauge transconjunctival vitrectomy. Mason et al. examined postoperative complications in 168 eyes of patients who had undergone sutureless 25-gauge vitrectomy for vitreous floaters. Cystoid macular edema and vitreous hemorrhage have been observed in some patients, but the rate of complications is low [17]. Yeh et al. assessed cases of 25-gauge transconjunctival sutureless vitrectomy for obtaining vitreous specimens in patients with intraocular lymphoma and reported the effectiveness of the technique for making a diagnosis, and they also reported a low rate of postoperative complications of 25-gauge transconjunctival sutureless vitrectomy for diagnosis and improvement in visual acuity compared to preoperative acuity [18].

In terms of the size of scars on the conjunctiva, 27-gauge vitrectomy has some advantages, since the sclerotomy is minimally invasive and less painful for patients [19]. However, there are fewer reports regarding the safety and efficacy of 27-gauge transconjunctival diagnostic vitrectomy. We therefore had a clinical interest in examining the benefits and complications associated with 27-gauge diagnostic vitrectomy, including that combined with cataract surgery. Therefore, the safety and effectiveness of 27-gauge diagnostic vitrectomy in a real-world setting at our hospital are reported.

## Materials and methods

A total of 32 eyes of 29 patients who underwent 27-gauge diagnostic vitrectomy surgery at the Osaka City University Hospital from July 2018 to December 2020 were included in the study. Data for these patients were examined retrospectively. All procedures performed in studies involving human participants were conducted in accordance with the ethical standards of the Institutional and/or National Research Committee and with the 1964 Declaration of Helsinki and its later amendments or comparable ethical standards.

Approval for this study was obtained prior to the start of the study from the Institutional Review Board at Osaka City University, Japan (IRB-4237). Written, informed consent for the storage of patient information in the hospital database and use in research was provided by all patients enrolled in the study.

The inclusion criteria for this study were as follows: (1) existence of inflammation or vitreous opacity; (2) no improvement of visual acuity, intraocular inflammation, or of the patient’s subjective symptoms with topical betamethasone and/or levofloxacin over 2 weeks; and (3) undergoing surgery with experienced vitrectomy surgeons (vitrectomy surgery > 2000 cases). The exclusion criterion was lack of written, informed consent.

The following data were collected: patient age and sex, best-corrected visual acuity (BCVA) preoperatively and 1 month and 6 months after surgery, duration of vitreous surgery, postoperative complications, and results of tests of vitreous specimens. All specimens underwent multiplex polymerase chain reaction (PCR) analysis for the presence of several different bacteria and viruses according to previously reported methods [9]. If patients were suspected of having intraocular lymphoma, concentrations of the cytokines interleukin IL-6 and IL-10 were determined, and gene arrangement studies (IgH arrangement) were conducted. An IL-10/IL-6 ratio > 1 strongly suggested a diagnosis of intraocular lymphoma [20].

Regarding visual acuity, the preoperative logarithm of the minimum angle of resolution (logMAR) visual acuity was compared with the 1-month, 3-month, and 6-month postoperative logMARs.

### Surgical procedure

All surgeries were performed under sub-tenon anesthesia consisting of 2% lidocaine with epinephrine and 0.25% bupivacaine. The Constellation Vision system (Alcon Laboratories, Inc., Fort Worth, TX, USA) was used as a 27-gauge transconjunctival sutureless pars plana vitrectomy system with a wide-angle noncontact viewing system (Resight®; Carl Zeiss Meditec AG, Jena, Germany). Surgeries were performed by three vitreoretinal surgeons (M.T., M.Y., S.H.) who had performed more than 2000 cases of micro-incision vitrectomy surgery (MIVS). After surgeons positioned the three trocars on the conjunctiva, a vitreous sample of approximately 1–2 ml was collected while the infusion was not open. The surgical parameters were 20,000 cuts per minute (cpm) and a vacuum of 0–650 mmHg. During surgery, intraocular pressure (IOP) was controlled to 15–20 mmHg with an IOP control system. Inner limited membrane peeling was performed in 11 cases (6 cases with phacoemulsification and intraocular lens implantation (PEA + IOL), 5 cases without PEA + IOL) using Brilliant Blue-G, and air tamponade was performed in 4 cases (2 cases with PEA + IOL, others without PEA + IOL). No scleral port sites were found to be leaking at the end of the surgery, so that all cases were without scleral suture.

A total of 16 of 32 eyes had cataracts, and since this can be problematic for vitrectomy, these eyes were treated with PEA and IOL.

### Statistical analyses

Collected data were entered into a Microsoft Excel spreadsheet (Microsoft, Redmond, WA) for further compilation and analysis. Statistical analyses were performed using Microsoft Excel, with a *p*-value < 0.05 considered significant. For the analyses, decimal visual acuity was converted to logMAR. When a patient’s decimal visual acuity was < 0.01, it was converted to logMAR according to the report of Johnson et al. [21].

## Results

### Preoperative characteristics

A total of 32 eyes of 29 patients underwent 3-port, 27-gauge pars plana diagnostic vitrectomy; there were 13 eyes of 12 male patients and 19 eyes of 17 female patients. The mean age of all 29 patients was 65.7 ± 14.9 (range 14–85) years, and the mean preoperative BVCA of all patients was 0.84 ± 0.87 logMAR. Of 16 of 32 eyes that underwent PEA + IOL, it was possible to follow 13 patients for 1 month, 11 patients for 3 months, and 8 patients for 6 months after the operation. Of 16 eyes without PEA + IOL, it was possible to follow 15 patients for 1 month, 9 patients for 3 months, and 7 patients for 6 months after the operation (Table 1).

**Table 1:** Preoperative characteristics of 32 patients who underwent 27-gauge diagnostic pars plana vitrectomy with/without PEA + IOL

	With PEA + IOL	Without PEA + IOL	All
Number of specimens	16	16	32
Age (years)	67.13 ± 10.14	64.19 ± 18.33	
Sex			
Male	9	4	13
Female	7	12	19
Preoperative visual acuity (logMAR)	0.9 ± 0.82	0.77 ± 0.91	
Preoperative diagnosis			
Intraocular lymphoma	3	4	7
Endophthalmitis	1	6	7
Asteroid hyalosis	1	0	1
Syphilitic uveitis	5	1	6
Lupus retinopathy	1	0	1
Ocular sarcoidosis	1	1	2
Chronic uveitis	1	2	3
HTLV-1–related uveitis	1	0	1
Acute retinal necrosis	2	0	2
Uveitis caused by *Toxoplasma*	0	1	1
Focal nodular gliosis	0	1	1

*MAR* Minimum angle of resolution

*PEA* Phacoemulsification and aspiration

*IOL* Intraocular lens implantation

### Results of diagnostic testing of vitrectomy specimens

The mean duration of all 3-port 27-gauge diagnosis vitrectomy operations was 40.1 ± 15.9 min. Based on whether PEA + IOL was conducted, the mean duration of vitrectomy with PEA + IOL was 46.4 ± 17.4 min, whereas the mean duration of vitrectomy without PEA + IOL was 34.8 ± 11.8 min (*p* = 0.04).

There were positive endogenous controls in all cases. All specimens were positive for the negative control in this test, so the validity of the test was confirmed. Nine patients had positive PCR results, as follows: Epstein-Barr virus (2 patients), *Treponema pallidum* (2 patients), bacterial 16-strip PCR (4 patients), human T-cell leukemia virus type-1 (1 patient), Varicella zoster virus (1 patient). Both *T. pallidum* and Epstein-Barr virus were found in the specimen of 1 patient.

In addition, 15 patients were tested for IL-6 and IL-10 levels to assess inflammatory status and diagnose lymphoma (Table 2).

**Table 2:** Concentrations of IL-6 and IL-10 in vitreous specimens taken from patients with suspected uveitis

Preoperative diagnosis	IL-6	IL-10
Intraocular lymphoma (*n* = 6)	69.1 ± 95.8	1311.7 ± 2387.6
Syphilitic uveitis (*n* = 3)	87.3 ± 20.3	8 ± 6.5
ARN (*n* = 2)	26,100 ± 500	380 ± 320
Uveitis caused by *Toxoplasma* (*n* = 1)	41.7	less than 2
HTLV-1–related uveitis (*n* = 1)	305	42
Chronic uveitis (*n* = 1)	860 /	less than 2
FNG (*n* = 1)	15.6	ー

*HTLV-1* Human T-cell leukemia virus type-1

*FNG* Focal nodular gliosis

*ARN* Acute retinal necrosis

Seven eyes of 5 patients were suspected of having intraocular lymphoma; the IL-10/IL-16 ratio was determined for 6 eyes of 5 patients, and 5 eyes of 4 patients were subjected to IgH arrangement analysis. In 4 eyes of 3 patients, the IL-6/IL-10 ratio was > 1; however, IgH arrangement was negative in 2 eyes, but the amount of the other specimens was too low for IgH arrangement analysis (these eyes were of the same patient). In patients in whom the IL-6/IL-10 ratio of the vitreous specimen was < 1, all IgH arrangement results were negative. In 1 patient, PCR testing of bacterial 16-strip was positive, and the IL-6/IL-10 ratio was also > 1 (IgH arrangement was negative). The diagnostic rate data are summarized in Table 3.

**Table 3:** Postoperative diagnostic rate and operative complications

	With PEA + IOL	Without PEA + IOL	All
Diagnostic rate	9/16 (56%)	8/16 (50%)	17/32 (53%)
Complication rate			
Vitreous hemorrhage	0	1/16 (6.3%)	1/32 (3.1%)
Increase in IOP	0	1/16 (6.3%)	1/32 (3.1%)

*PEA* Phacoemulsification and aspiration

*IOL* Intraocular lens implantation

*IOP* Intraocular pressure

With regard to postoperative complications, 1 patient had increased (IOP after vitrectomy and was treated with dorzolamide and timolol maleate eye drops. There was also 1 patient with vitreous hemorrhage and decreased IOP; this patient underwent re-operation with 27-gauge vitrectomy, and the vitreous fluid was replaced with air.

### Visual acuity

Postoperative mean BCVA improved to 0.42 ± 0.55 logMAR at 1 month (*p* = 0.004, *n* = 28), 0.47 ± 0.61 logMAR at 3 months (*p* = 0.003, *n* = 20), and 0.46 ± 0.73 logMAR at 6 months (*p* = 0.011, *n* = 15). In patients with PEA + IOL, mean BCVA improved to 0.37 ± 0.33 logMAR at 1 month (*p* = 0.03, *n* = 13), 0.39 ± 0.38 logMAR at 3 months (*p* = 0.02, *n* = 11), and 0.38 ± 0.31 logMAR at 6 months (*p* = 0.06, *n* = 8). Of the patients who underwent diagnostic vitrectomy without PEA + IOL, mean BCVA improved to 0.43 ± 0.69 logMAR at 1 month (*p* = 0.06, *n* = 15), 0.57 ± 0.80 logMAR at 3 months (*p* = 0.051, *n* = 9), and 0.45 ± 1.00 logMAR at 6 months (*p* = 0.09, *n* = 7). Comparing these two groups in terms of visual acuity, there was no significant difference at any point (*p* = 0.67 preoperatively, *p* = 0.79 at 1 month, *p* = 0.53 at 3 months, and *p* = 0.86 at 6 months after surgery), as summarized in Table 4.

**Table 4:** Postoperative BCVA comparing vitrectomy with PEA + IOL and vitrectomy without PEA + IOL

Preoperative BCVA (logMAR)	0.84 ± 0.87 (of all patients)	
	With PEA + IOL	Without PEA + IOL
Preoperative BCVA (logMAR)	0.90 ± 0.82	0.77 ± 0.91
Postoperative BCVA (logMAR)		
1 month later	0.37 ± 0.34 (*p* = 0.03)	0.43 ± 0.68 (*p* = 0.06)
3 months later	0.39 ± 0.38 (*p* = 0.02)	0.57 ± 0.80 (*p* = 0.051)
6 months later	0.38 ± 0.31 (*p* = 0.06)	0.45 ± 1.00 (*p* = 0.09)

*BCVA* Best corrected visual acuity

*PEA* Phacoemulsification and aspiration

*IOL* Intraocular lens implantation

*MAR* Minimum angle of resolution

## Discussion

Diagnostic vitrectomy is a very common and important clinical option for diagnosing uveitis, including primary intraocular lymphoma [18, 22, 23]. Some case reports have described the diagnostic rate of MIVS, including 27-gauge pars plana vitrectomy [24, 25]. As far as we know, this case series is the first report describing 27-gauge vitrectomy for the diagnosis of uveitis. Diagnostic and complication rates were acceptable even when compared with other gauges, including other MIVS in previous articles (Table 5).

**Table 5:** Summary of diagnostic vitrectomy in the current and previous studies

	Year	Size of gauge in vitrectomy	Specimens or patients	Diagnostic rate	Complications	Preoperative visual acuity	Postoperative visual acuity
Current study	2021	27	32	17/32 (53.1%)	vitreous hemorrhage: 1, increased IOP: 1	0.77 ± 0.91 (without PEA + IOL, logMAR)	0.43 ± 0.68 (without PEA + IOL, logMAR, *p* = 0.057)
Wittenberg et al [14]	2008	20	228	126/228 (55.3%)	Not mentioned	not mentioned	not mentioned
Davis et al [22]	2005	20	78	48/78 (61.5%)	RD: 5, gla: 4, cat: 8/48 CME: 5	Median: 20/200, range: 20/20—LP	Median: 20/75, range: 20/20—LP
Mruthyunjaya	2002	20	90	35/90 (38.8%)	Not mentioned	Not mentioned	Not mentioned

*Cat* Cataract, *CME* Cystic macular edema, *gla* Glaucoma, *RD* Retinal detachment

In recent years, MIVS has shown remarkable progress, and the application of 27-gauge pars plana vitrectomy has become widespread, especially in Japan, with good clinical results to date [26, 27]. The present results indicate that diagnostic vitrectomy is very safe, and the frequency of complications is low, even in cases of uveitis. The low surgical complication rate of the 27-gauge system and its safety have already been reported in several studies [28–30]. In the present study, there were originally postoperative complications due to the activity of the uveitis, but there were no complications due to the surgical operation itself.

**Fig. 1:**
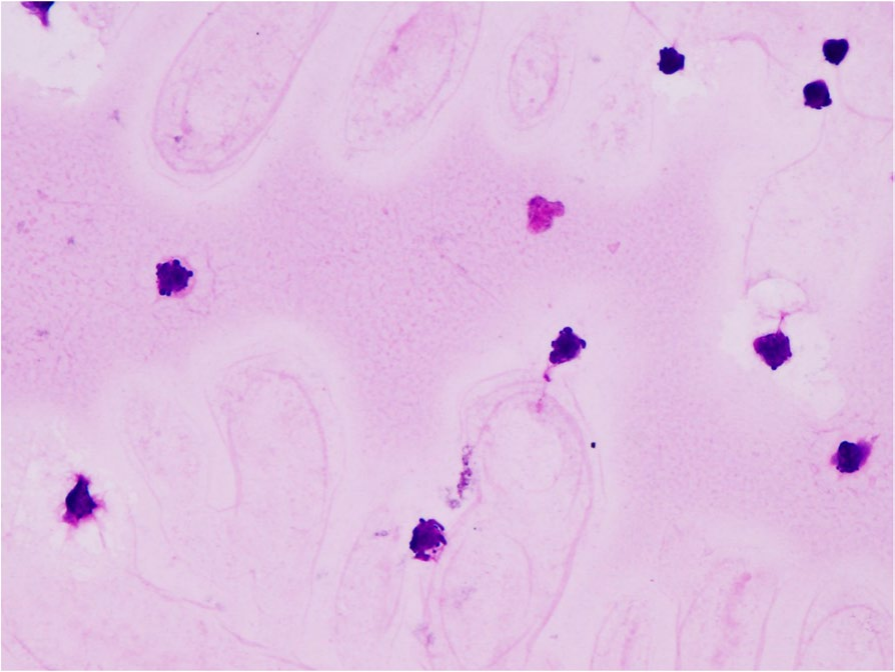
Cytological photograph (flower-like cells) of vitreous sample from an HTLV-1 uveitis patient (× 400)

In addition, there was a possibility that the vitreous humor sample would be crushed when the MIVS became a small gauge, but it was possible to obtain a morphologically diagnosable sample by cytology in an HTLV-1 uveitis patient (Fig. 1). Table 5 summarizes previous reports describing 20-gauge and small-gauge techniques.

For uveitis of unknown cause, multiplex PCR is an established method for excluding an infectious origin. In particular, as reported by Sugita et al. and Nakano et al., the probability of diagnosis if infection is present is extremely high [7–9]. Not all of the present cases were strongly suspected of having an infectious etiology, but testing as an exclusionary diagnosis and positive endogenous control in all cases indicated that 27-gauge diagnostic vitrectomy is useful as a basic test.

In addition, the levels of IL-10 and IL-6 were investigated in cases suspected of having intraocular lymphoma. In addition, determining IL-6 levels in other cases of uveitis as well enables evaluation of the extent of intraocular inflammation and could thus prove beneficial in selecting treatment options such as tocilizumab [31, 32]. Furthermore, the improvement in visual acuity is only a secondary effect. This is because, in the combination cataract surgery cases, it could be caused by the improvement of the cataract, and it depends more on the pathological condition of uveitis itself than on the effect of vitrectomy in the first place, so that using visual acuity as a parameter may have been a confounding factor in this study.

In intraocular lymphoma cases, flow cytometry, cytokine evaluation, gene rearrangement studies, and cooperation with an ophthalmic pathologist are indispensable to reach an accurate diagnosis [11, 33–36]. Such patients are often diagnosed with uveitis of unknown cause and treated with long-term steroids; however, their symptoms worsen [37]. In terms of clinical diagnostic effectiveness, some studies have reported success with vitrectomy. Wittenberg et al., Davis et al., and Mrunthunjaya et al. reported diagnostic rates of 55.3%, 61.5%, and 38.8%, respectively, for 20-gauge diagnostic vitrectomy [14, 15, 22]. The present result was with a minimally invasive 27-gauge, with almost the same diagnostic accuracy as with 20-gauge, and there were not many complications (complication rate: 6.4% (2/31)).

The primary limitations of the present study include its small case series design (*N* = 31) and that it was a cohort study with no comparison group. In addition, the effect of improving visual acuity is more limited because it is related to the pathological condition of uveitis rather than the effect of vitrectomy. Another limitation of this study is the fact that all cases were not reviewed by an ocular pathologist. If they had been, the accuracy of diagnosis may have increased, and 27-gauge was not compared with the other MIVS systems in this case series.

In conclusion, the results of the present study are useful for diagnosing intraocular lymphoma and uveitis of unknown origin. Furthermore, the extent of inflammation can be estimated by measuring the levels of cytokines such as IL-6 using specimens obtained via minimally invasive 27-gauge vitrectomy. Notably, perioperative complications are the same as previously reported. Thus, 27-gauge diagnostic vitrectomy was helpful in the management of posterior uveitis or panuveitis of unknown origin.

## Data Availability

The data generated and/or analyzed for this study are available from the corresponding author upon request.
